# The citrus flavonoid naringenin impairs the *in vitro* infection of human cells by Zika virus

**DOI:** 10.1038/s41598-019-52626-3

**Published:** 2019-11-08

**Authors:** Allan Henrique Depieri Cataneo, Diogo Kuczera, Andrea Cristine Koishi, Camila Zanluca, Guilherme Ferreira Silveira, Thais Bonato de Arruda, Andréia Akemi Suzukawa, Leandro Oliveira Bortot, Marcelo Dias-Baruffi, Waldiceu Aparecido Verri, Anny Waloski Robert, Marco Augusto Stimamiglio, Claudia Nunes Duarte dos Santos, Pryscilla Fanini Wowk, Juliano Bordignon

**Affiliations:** 1 Laboratório de Virologia Molecular, Instituto Carlos Chagas/Fiocruz-PR, Curitiba, Paraná Brazil; 2 Laboratório de Física Biológica, Departamento de Física e Química, Faculdade de Ciências Farmacêuticas, Universidade de São Paulo, Ribeirão Preto, São Paulo Brazil; 3 Laboratório de Glicoimunologia, Departamento de Análises Clínicas, Toxicológicas e Bromatológicas, Faculdade de Ciências Farmacêuticas, Universidade de São Paulo, Ribeirão Preto, São Paulo Brazil; 4 Departamento de Ciências Patológicas, Centro de Ciências Biológicas, Universidade Estadual de Londrina, Paraná, Brazil; 5 Laboratório de Células Tronco, Instituto Carlos Chagas/Fiocruz-PR, Curitiba, Paraná Brazil

**Keywords:** Antivirals, Viral infection

## Abstract

The Zika virus (ZIKV) is an arthropod-borne virus that belongs to the *Flaviviridae* family. The ZIKV infection is usually asymptomatic or is associated with mild clinical manifestations; however, increased numbers of cases of microcephaly and birth defects have been recently reported. To date, neither a vaccine nor an antiviral treatment has become available to control ZIKV replication. Among the natural compounds recognized for their medical properties, flavonoids, which can be found in fruits and vegetables, have been found to possess biological activity against a variety of viruses. Here, we demonstrate that the citrus flavanone naringenin (NAR) prevented ZIKV infection in human A549 cells in a concentration-dependent and ZIKV-lineage independent manner. NAR antiviral activity was also observed when primary human monocyte-derived dendritic cells were infected by ZIKV. NAR displayed its antiviral activity when the cells were treated after infection, suggesting that NAR acts on the viral replication or assembly of viral particles. Moreover, a molecular docking analysis suggests a potential interaction between NAR and the protease domain of the NS2B-NS3 protein of ZIKV which could explain the anti-ZIKV activity of NAR. Finally, the results support the potential of NAR as a suitable candidate molecule for developing anti-ZIKV treatments.

## Introduction

The Zika virus (ZIKV) is an arthropod-borne virus from the *Flavivirus* genus and the *Flaviviridae* family that was first isolated from a rhesus monkey from the Zika forest of Uganda in 1947^1^. The ZIKV is transmitted primarily by *Aedes spp*. mosquitoes^2^. However, transmission by sexual contact, via contaminated blood and from mother to fetus has also been described^3–5^.

Infection by ZIKV progresses as a self-limiting disease like observed in dengue infection, with mild clinical manifestations such as fever, macular or papular rash, arthritis and arthralgia, nonpurulent conjunctivitis, myalgia, headache, edema, orbital pain and vomiting^6,7^. However, during Zika epidemics in French Polynesia (2013) and Brazil (2015), greater numbers of cases of *Guillain-Barré* and congenital syndrome (microcephaly) were observed^7–9^. Strong evidence suggests a link between ZIKV infection and microcephaly once the viral genome has been detected in the amniotic fluid and brains of affected fetuses^5,9^.

In Brazil, between December 2015 and March 2018 more than 230,000 cases of ZIKV-infection were reported^10^. Additionally, 3,100 cases of children with ZIKV-associated congenital and neurological syndrome were confirmed^11^. At present, neither a vaccine nor an antiviral drug is available to prevent or treat the ZIKV infection. Despite recent advances in drug discovery against ZIKV, no antiviral compound has been authorized at the phase I clinical trial level^12^. Flavonoids are polyphenolic compounds that are present in a wide range of fruits and vegetables as low-molecular weight secondary metabolites^13^. Flavonoids are known to have antiviral activity against HIV-1, herpes simplex 1 and 2, influenza, dengue and yellow fever^14–18^. Additionally, it was demonstrated that several flavonoids could inhibit Zika virus infection *in vitro* and *in vivo*^19–25^. The antiviral effect of flavonoids seems to occur through interactions between the phenol rings of flavonoids and viral proteins and/or RNA, or via its capacity to interfere in host cell defense by regulating MAP kinase signaling^26–29^. Naringenin (NAR) (4, 5, 7-trihydroxyflavanone), a natural flavonoid aglycone of naringin, is widely distributed in citrus fruits, tomatoes, cherries, grapefruits and cocoa^30^. NAR was shown to present a wide range of activities, including anti-inflammatory and analgesic actions^30–32^. In addition, NAR was recently shown to have anti-dengue virus activity *in vitro*^18^, and the similarities between these flaviviruses prompted us to test NAR against ZIKV infection *in vitro*.

Here, NAR was shown to present *in vitro* anti-ZIKV activity against four different strains of recent clinical isolates of the Asian lineage and one classical African lineage. Additionally, the antiviral activity of NAR seems to occur in the late steps of virus life cycle. A promising characteristic of NAR is that the antiviral effect is observed even when it is added to cultures 24 hours after the establishment of the infection. Finally, *in silico* docking analysis suggests a close interaction between NAR and the protease domain of ZIKV, strengthening the data from *in vitro* assays.

## Results

### High doses of NAR affect A549 cell viability

**Figure 1:**
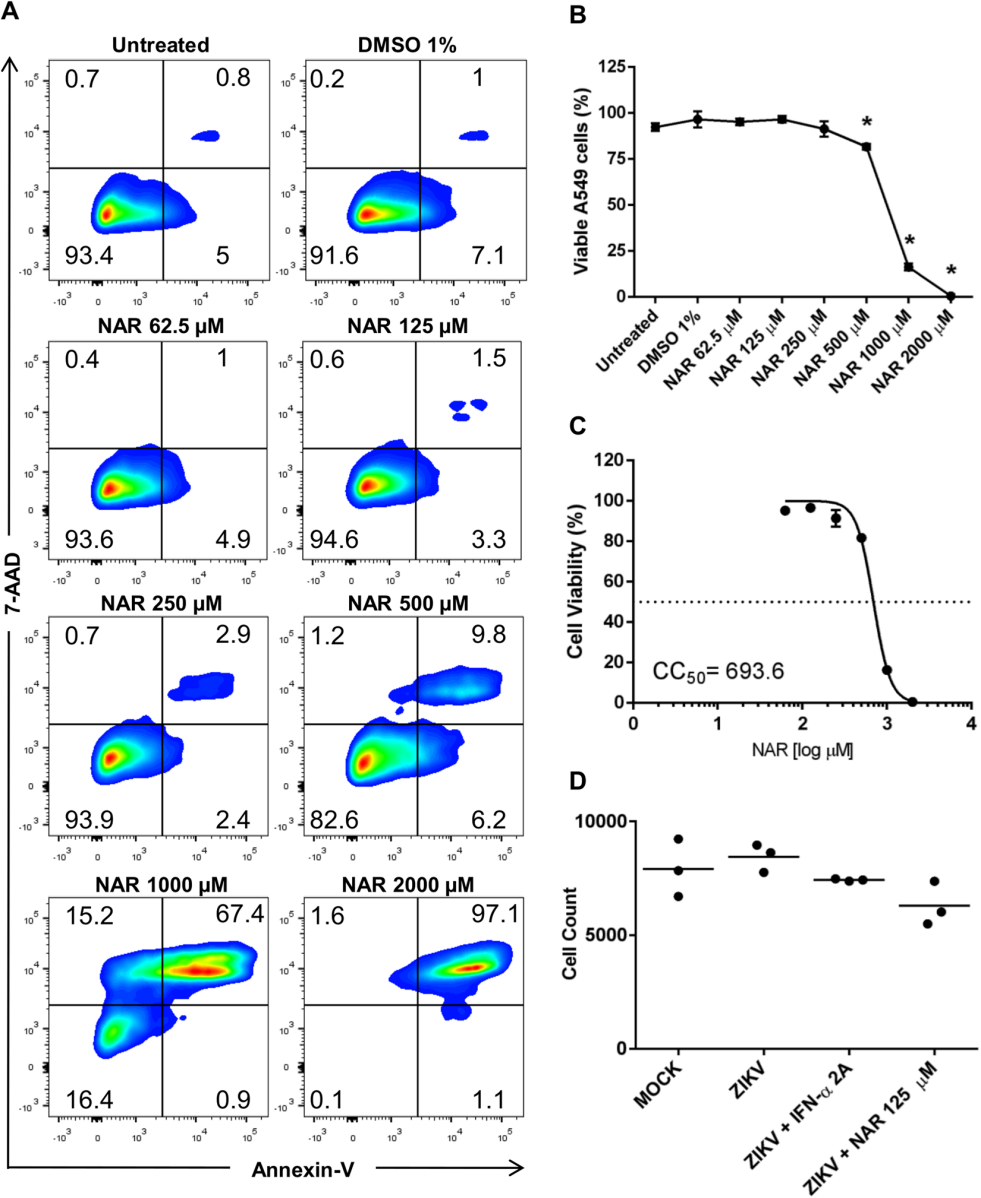
Toxicity of NAR to A549 cells. A549 cells were treated with different concentrations of NAR (2,000 to 62.5 μM) for 48 hours. DMSO (1%) was used as a control and vehicle for the preparation of NAR stock. The cell viability was analyzed through Annexin-V and 7-AAD staining by flow cytometry. (**A**) Representative density plot showing the Annexin-V and 7-AAD staining of one representative experiment. (**B**) Viable A549 cells (Annexin-V^−^/7-AAD^−^). (**C**) The NAR concentration that promoted a 50% reduction in cell viability (CC_50_) was obtained by using nonlinear regression, a sigmoidal concentration-response curve and a variable slope (GraphPad Prism; La Jolla, CA.USA). (**D**) The average number of cells (cell nuclei count after counterstained with DRAQ5) presented in culture in each experimental condition determined by Operetta high-content imaging system. Analyses were performed using a one-way ANOVA followed by Tukey´s Multiple Comparison Test (*p < 0.05 *vs* untreated). All the data represent three independent experiments, each one in technical triplicate.

To determine the concentration of NAR to use for the *in vitro* assays, we performed a viability assay. Results indicated that NAR is toxic for A549 cells at higher concentrations (from 500 to 2,000 μM) (Fig. 1A,B). After treatment of A549 cells with 500 μM of NAR 10% of cells become apoptotic (Fig. 1A,B). Also, data demonstrated, using double-negative A549 cells (Annexin-V^−^/7-AAD^−^), that NAR could be safely used in the antiviral assays (Fig. 1A,B). Moreover, double-negative A549 cells were used to establish the cytotoxic concentration for 50% of the culture (CC_50_), which was calculated as 693.6 μM (Fig. 1C). Despite 250 μM NAR being non-toxic for A549 cells (Fig. 1A,B), it was defined 125 μM of NAR as the maximal non-toxic concentration (MNTC), to avoid residual toxicity and with the aim of using a lower concentration of NAR in the antiviral assays. Moreover, the cell nuclei count using Operetta high-content imaging show that NAR (125 μM) did not reduce the number of cells compared to untreated A549 cells (Fig. 1D). Altogether, results from different assays confirm that 125 μM of NAR was safe to use in A549 cells and did not show toxic effects.

### *In vitro* inhibition of Zika virus infection by NAR is concentration-dependent

**Figure 2:**
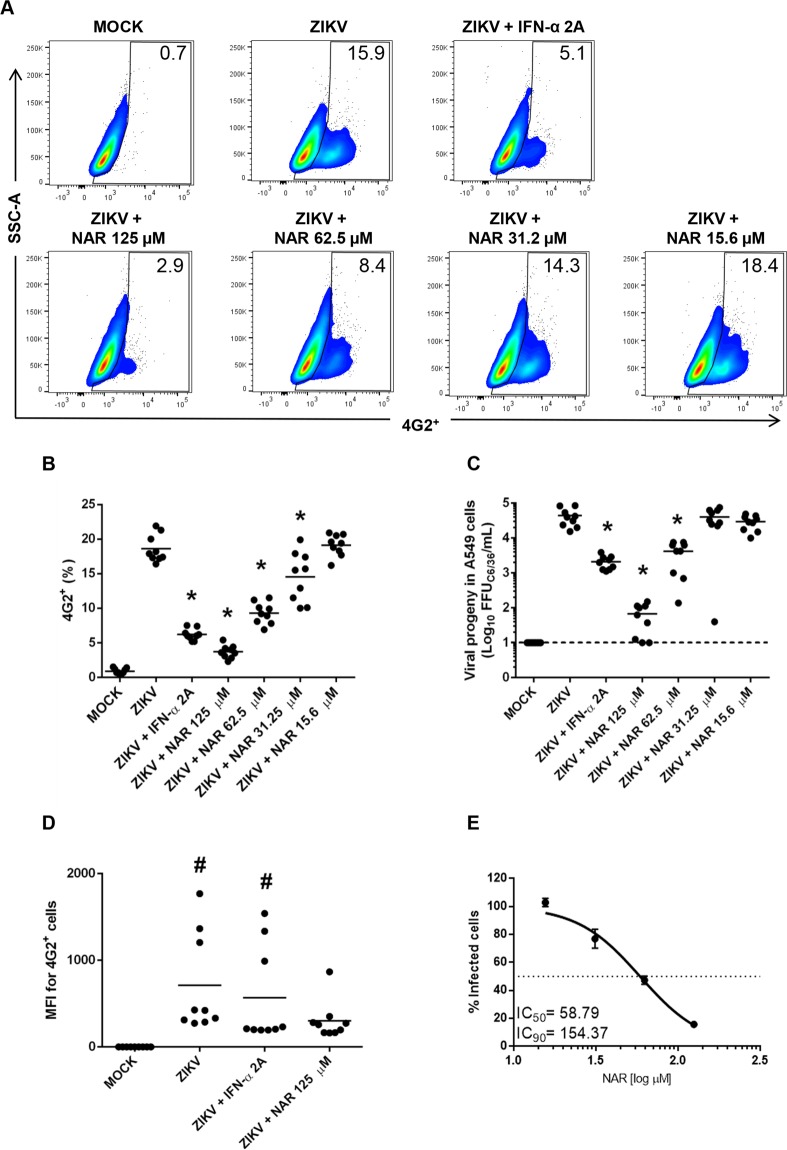
Anti-Zika virus activity of NAR is concentration-dependent. The A549 cells were infected with ZIKV (ZV BR 2015/15261; MOI 0.1) for 90 minutes and treated with different concentrations of NAR (125 to 15.6 µM) after inoculum removal. After 48 hours, the cells were harvested and stained for flow cytometry assay using anti-flavivirus E protein monoclonal antibody (4G2). IFN-α 2A (200 IU/mL) was used as a positive anti-viral control, and the non-infected cells (MOCK) were used as a negative control. (**A**) Representative density plot showing the frequency of A549-infected cells (4G2^+^) after treatment with different concentrations of NAR. (**B**) The frequency of ZIKV-infected A549 cells (4G2^+^) after treatment with NAR. (**C**) The viral titers detected by foci-forming immunodetection assay (FFU_C6/36_/mL) in A549 cell culture supernatant after 48 hours of infection. (**D**) The quantification of the mean fluorescence intensity (MFI) in 4G2^+^ cells. (**E**) The concentration response curve of NAR against ZIKV. The NAR concentration that inhibited 50% and 90% of the infection (IC_50_ and IC_90_) was defined using a sigmoidal dose response curve (variable slope). Results from three independent experiments performed in technical triplicate and analyzed by one-way ANOVA followed by Tukey´s Multiple Comparison Test (*p < 0.05 *vs* ZIKV infected and untreated cells and # *p* < 0.05 *vs* mock-infected cells). The dashed line represents the assay’s limit of detection.

Based on the previous data showing the anti-dengue virus activity of NAR^18^, a set of experiments were performed to evaluate the anti-ZIKV activity of NAR. A549 cells were infected with ZIKV (ZV BR 2015/15261) and treated with different concentrations of NAR (15.6, 31.25, 62.5 and 125 µM) after inoculum removal. According to the literature^33^ and kinetic experiments it was observed that 48 h was the best time point for immunostaining once at 72 h we observe cytopathic effect (Supplementary Fig. 1). Thus, to access antiviral activity of NAR, FACS and the foci-forming immunodetection assay were performed in cells and cell culture supernatant after 48 hours post-infection (hpi), respectively. Results demonstrate that the anti-ZIKV activity of NAR was concentration-dependent (Fig. 2A–C). Surprisingly, IFN-α 2A-treated cells presented more viable ZIKV particles in the cell culture supernatant than NAR-treated cells did (Fig. 2C). An analysis of the mean fluorescence intensity (MFI) of infected cells indicates that IFN-α 2A controls the number of infected cells; however, the production of the virus is not reduced to the same level compared to NAR-treated cells (Fig. 2D). Additionally, the IC_50_ and IC_90_ indexes were calculated as 58.79 and 154.37 µM, respectively, with confidence of interval of 95% (Fig. 2E). The selective index (CC_50_/IC_50_) of NAR was 11.79.

**Figure 3:**
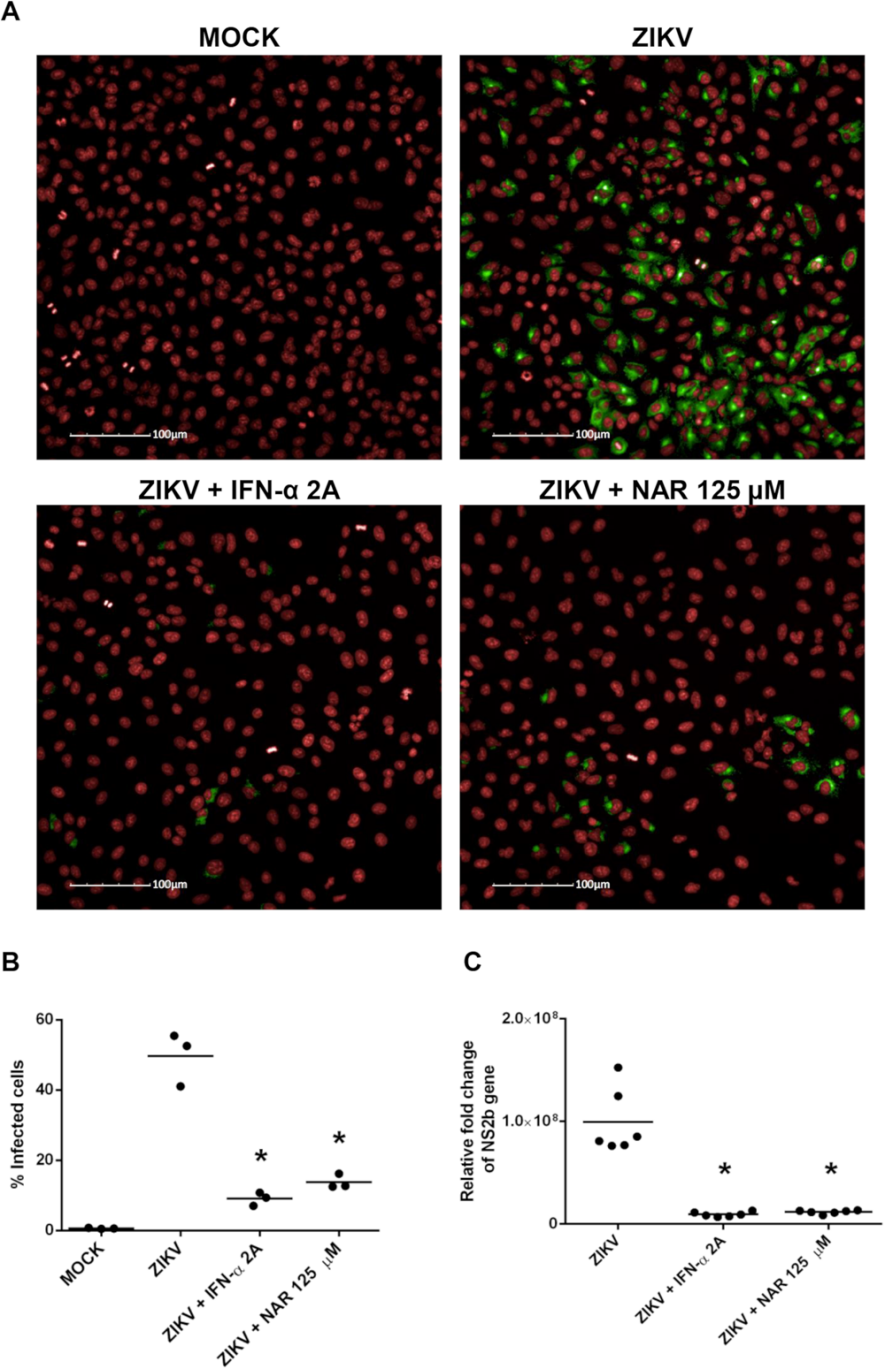
NAR impairs the A549 infection by Zika virus. A549 cells were infected with ZIKV (ZV BR 2015/15261; MOI 0.1) and treated with NAR (125 µM) or IFN-α 2A (200 IU/mL) after inoculum removal or uninfected (MOCK-control). After 48 hours, the numbers of infected A549 cells were quantified using a high-content imaging assay (20X magnification; Operetta High-Content Imaging System from PerkinElmer). (**A**) Representative imaging of A549 cells obtained by the Operetta System showing red cell nuclei (counterstained with DRAQ5), and in green anti-flavivirus E protein monoclonal antibody (4G2) plus goat anti-mouse Alexa Fluor 488 secondary antibody. (**B**) The average number of infected-A549 cells. (**C**) ZIKV RNA detection in A549 cells by RT-qPCR assay. The ZIKV NS2B RNA was quantified in infected cells compared to mock-infected cells (relative fold change) and normalized to the housekeeping gene *18S*. Results from three independent experiments, in technical triplicate (except the RT-qPCR, which was performed in technical duplicate), were analyzed by one-way ANOVA followed by Tukey´s Multiple Comparison Test (*p < 0.05 *vs* ZIKV*-*infected cells).

Using an immunofluorescence assay (Operetta high-content imaging), NAR was shown to reduce the number of A549-infected cells (Fig. 3A,B). Additionally, RT-qPCR (Fig. 3C) demonstrated that NAR impairs ZIKV replication. Furthermore, results from two different assays indicate the anti-ZIKV activity of NAR was not due to a virucidal effect on the virus particles (Supplementary Fig. 2). Virus particles treated with NAR (125 µM) did not affect viral infectivity, suggesting no effect on the viral structure (Supplementary Fig. 2). Additionally, it was tested if NAR treatment (125 µM) of A549 cells could modulate cell autofluorescence. Using FACS it was demonstrated that NAR does not affected A549 cells autofluorescence compared to untreated cells (Supplementary Fig. 3).

### Anti-Zika virus activity of NAR is lineage-independent

**Figure 4:**
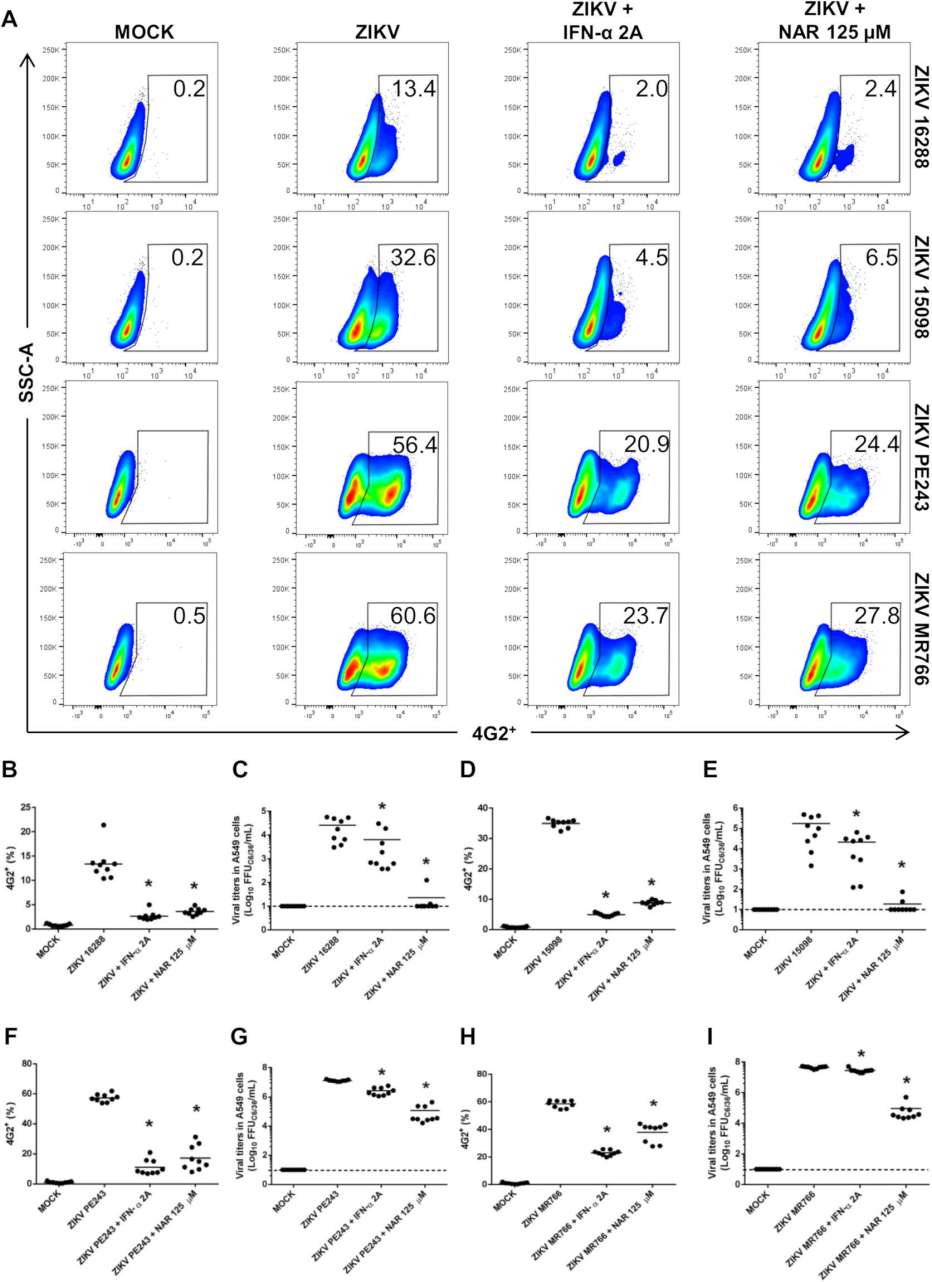
NAR impairs the infection of Zika virus from Asian- and African-lineage in A549 cells. A549 cells were infected with four additional strains of ZIKV (ZV BR 2016/16288, ZV BR 2015/15098, ZIKV PE243, ZIKV MR766; MOI 0.1), three from Asian- and one from African-lineage and treated with NAR (125 μM) or IFN-α 2A (200 IU/mL). After 48 hours, the cells were harvested and stained for flow cytometry assay using anti-flavivirus E protein monoclonal antibody (4G2). (**A**) A representative density plot showing the frequency of A549 cells (4G2^+^) that were infected with the ZV BR 2016/16288, ZV BR 2015/15098, ZIKV PE243 and ZIKV MR766 strains. The frequency of A549 cells infected (4G2^+^) with the ZV BR 2016/16288 (**B**), ZV BR 2015/15098 (**D**), ZIKV PE243 (**F**) and ZIKV MR766 (**H**) strains. Determination of viable ZIKV in the cell culture supernatant of A549 cells infected with ZV BR 2016/16288 (**C**), ZV BR 2015/15098 (**E**), ZIKV PE243 (**G**) and ZIKV MR766 (**I**) strains using the foci-forming immunodetection assay (FFU_C6/36_/mL). The data represent three independent experiments, each one in technical triplicate then analyzed by a one-way ANOVA followed by Tukey´s Multiple Comparison Test (*p < 0.05 *vs* ZIKV infected and untreated A549 cells).

Using phylogenetic analyses it was demonstrated the existence of two main Zika virus lineages, the African- and Asian- lineages^34^. To confirm the Asian- and African-origin of the virus a real time RT-PCR assay was performed using a set of primers and probes employed as previously described^35,36^ (Supplementary Table 1 and Supplementary Fig. 4). Moreover, it was shown that the African- and Asian-lineage of ZIKV present differences in virulence/pathogenicity *in vitro* and *in vivo*^37^. Thus, to exclude a lineage-specific anti-ZIKV activity of NAR against ZV BR 2015/15261, four additional ZIKV strains were tested, three from Asian-lineage (ZV BR 2016/16288, ZV BR 2015/15098, ZIKV PE243) and one from African-lineage (ZIKV MR766). Using FACS and the foci-forming immunodetection assay, it was possible to demonstrate that NAR impairs the A549-infection with both Asian- and African-lineages of ZIKV (Fig. 4). However, despite NAR was effective to reduce the infection with both ZIKV lineages, a higher effect was observed for Asian-lineage (~4 fold reduction of infection) when compared to African-lineage (~2 fold reduction of infection) (Supplementary Fig. 5).

### Time of NAR addition experiments

**Figure 5:**
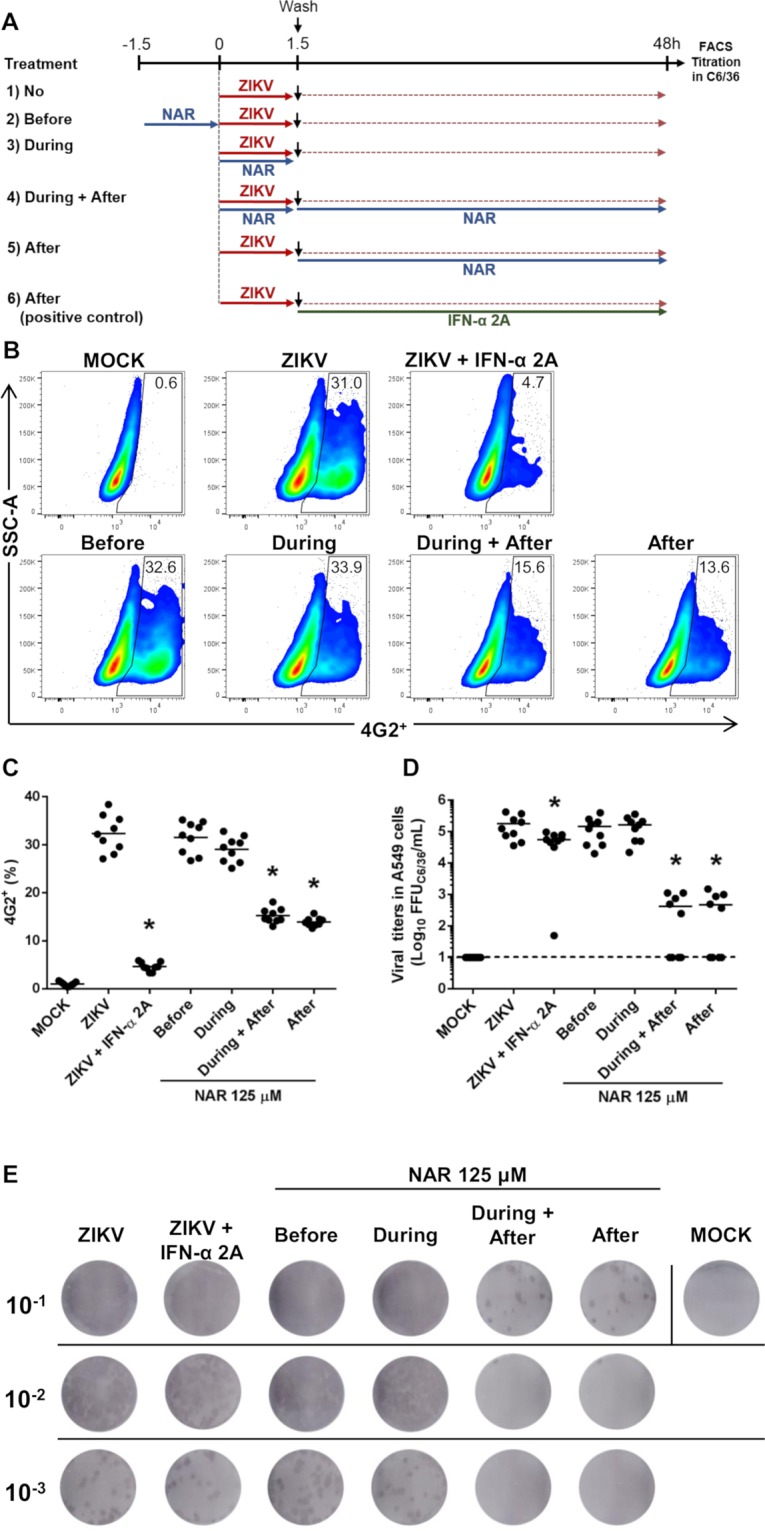
Time-of-drug-addition experiments of NAR in A549 cells. The A549 cells were infected with 0.1 MOI of ZIKV (ZV BR 2015/15261 strain) and cells were treated with NAR (125 μM) at different moments. (**A**) Schematic representation from the experimental design. A549 cells were submitted to different treatment regimens: (1) infected and left untreated (positive control of infection); (2) treated with NAR (125 μM) for 90 minutes prior to ZIKV infection; (3) treated with NAR during ZIKV infection; (4) treated with NAR during and after ZIKV infection; (5) treated with NAR after ZIKV infection; or (6) treated with IFN-α 2A (200 UI/ml) after ZIKV infection as a control of treatment. (**B**) Representative density plot showing the frequency of infected cells (4G2^+^) in each experimental condition. (**C**) Frequency of ZIKV-infected cells (4G2^+^). (**D**) The viral titers detected by foci-forming immunodetection assay (FFU_C6/36_/mL) from cell culture supernatant. (**E**) Representative foci-forming immunodetection assay (FFU_C6/36_/mL) from Fig. 5D. The data represent three independent experiments in technical triplicate that were analyzed by one-way ANOVA followed by Tukey´s Multiple Comparison Test (*p < 0.05 *vs* ZIKV-infected and untreated cells).

**Figure 6:**
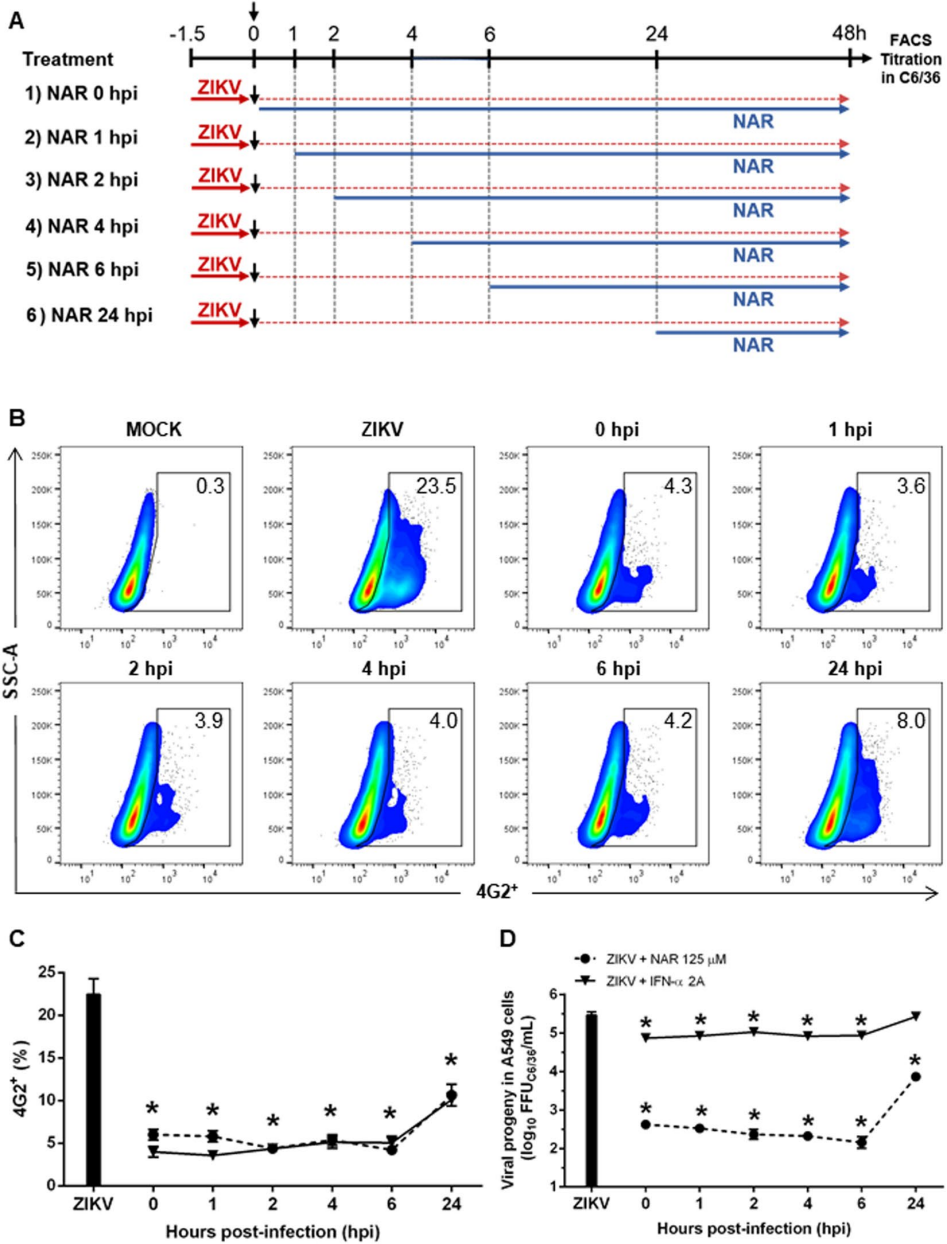
Naringenin treatment was effective even after the infection establishment. The A549 cells were infected with ZV BR 2015/15261 (MOI 0.1) and treated (one time) with NAR (125 μM) or IFN-α 2A (200 IU/mL) at different times points after the infection establishment, at 0, 1, 2, 4, 6, and 24 hours post-infection (hpi). After 48 h of infection, the A549 cells were harvested and stained with mouse anti-flavivirus E protein (4G2) monoclonal antibody for the flow cytometry analyses. (**A**) Schematic representation of the experimental design. (**B**) Representative density plot showing the frequency of A549-infected cells (4G2^+^). (**C**) Frequency of ZIKV-infected A549 cells (4G2^+^). (**D**) The viral titers detected by foci-forming immunodetection assay (FFU_C6/36_/mL) in the supernatant of A549-infected cells. The dotted line represents A549 cells treated with NAR, and the continuous line represents A549 treated with recombinant IFN-α 2A. The data represent three independent experiments for which each was performed in technical triplicate then analyzed by one-way ANOVA followed by Tukey´s Multiple Comparison Test (*p < 0.05 *vs* ZIKV-infected and untreated cells).

To determine which step of the ZIKV life cycle NAR exerts its effects, a time of addition experiment was performed in A549 cells^18,38^ (Fig. 5A). The results indicate that NAR treatment simultaneous to the addition of ZIKV-inoculum and after inoculum removal (during + after infection) or only after inoculum removal (after infection) seems to impact the ZIKV infected-A549 cells (Fig. 5B–E). Also, a slightly anti-ZIKV effect was observed if A549 cells were pretreated with NAR for long periods of time (18 h) before infection, compared to 1.5 h used in the experiment (Supplementary Fig. 6). Additionally, to confirm the efficiency of NAR treatment, we added NAR to the cell cultures at different time points after infection establishment (Fig. 6A–D). The results showed the reduction of viral titers in A549 cells even when cells were treated 24 hpi. However, when the NAR treatment was postponed to 24 hpi, we observe increased viral titers in cell culture supernatant when compared to those observed in A549 cells treated up to 6 hpi. Otherwise, IFN-α 2A showed similar results for the reduction of infected cells, but it was not as efficient at reducing the viable virus particles in the cell culture supernatant as NAR (Fig. 6D). Taken together, these results suggest that NAR might act on the replication, maturation or assembly of viral particles.

### Molecular docking of NAR

*In silico* analyzes, as molecular docking has been used to indicate the potential mechanism of action of flavonoids against Zika virus^24,39^. Once the experimental data indicated that NAR could act on the replication, maturation or assembly of viral particles, we employed molecular modeling to assess the hypothesis that NAR may act as a non-competitive inhibitor of the NS2B-NS3 viral protease. First, we validated a docking protocol using the available experimental data^28^. For validation, two flavonoids known as non-competitive inhibitors of the ZIKV protease were chosen, namely myricetin, because of its high activity, and apigenin, due to its close structural similarity to NAR^27^. Docking calculations show that NAR is able to interact with the ZIKV protease in a similar way as myricetin and apigenin (Supplementary Fig. 7). Thus, we speculate that NAR is able to bind to the ZIKV protease and inhibit it via a non-competitive mechanism in the same way as other flavonoids. Experimental validation is needed to prove the interaction between NAR and ZIKV-protease.

### Anti-ZIKV activity of NAR in different cell lines

As ZIKV-infection could trigger neurological defects in children born from infected women^5,9,40^, the *in vitro* anti-ZIKV activity of NAR was assessed in cell lines potentially involved in ZIKV-pathogenesis, like human glioblastoma cell line (A172) and human embryonic stem cell line (NKX2-5^eGFP/w^hESC). Thus, using ZIKV strains from Asian- and African-lineage and two different techniques (FACS and foci-forming immunodetection assay) it was shown that NAR was able to reduce the infection in glioblastoma cell line (A172) (Supplementary Fig. 9B), while no effect was observed in human embryonic stem cell line (Supplementary Fig. 8). In spite of the fact that no effect was observed in human embryonic stem cell line, the anti-ZIKV activity of NAR was confirmed using other human cell line, Huh7.5, from the hepatic origin (Supplementary Fig. 9D).

**Figure 7:**
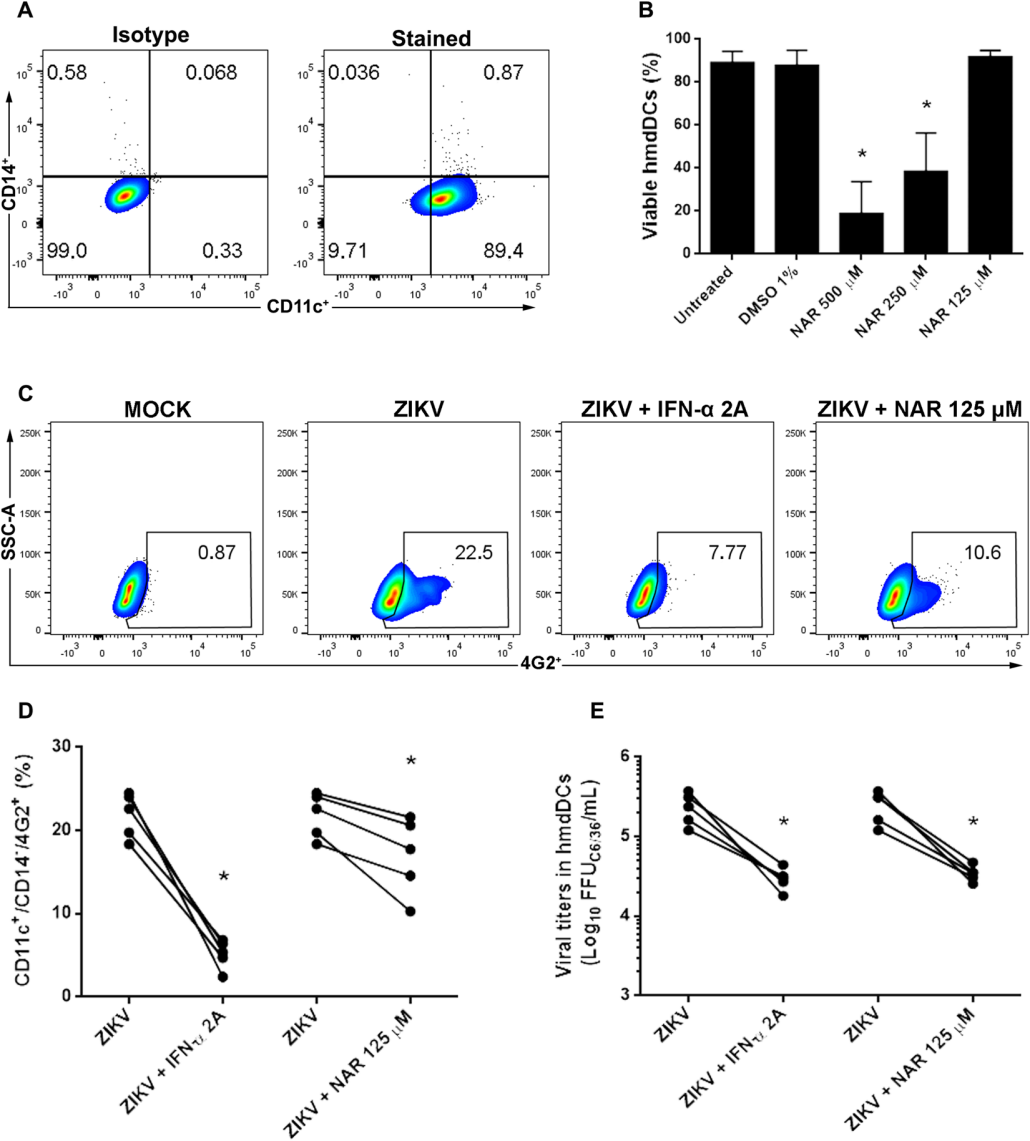
NAR impairs the infection of human monocyte-derived dendritic cells by ZIKV. Human monocytes derived-dendritic cells from five healthy donors were tested. (**A**) Representative density plot showing the phenotype of hmdDCs (CD14^−^/CD11c^+^) after seven days of differentiation. To determine the MNTC of NAR for hmdDCs cells were treated with different concentrations of NAR (500 to 125 µM) for 24 h. DMSO was used as a control and vehicle for the preparation of NAR stock solution. (**B**) The frequency of viable hmdDCs (Annexin-V^−^/7-AAD^−^) were shown. For antiviral activity of NAR, hmdDCs were infected with ZIKV (ZV BR 2015/15261 at MOI 10) and treated with NAR (125 μM) or IFN-α 2A (200 IU/mL). After 24 hours, the cells were harvested and stained for flow cytometry assay using anti-human CD11c-PE, anti-CD14-APC and anti-flavivirus E protein monoclonal antibody (4G2-FITC). (**C**) Representative density plot showing the frequency of hmdDCs-infected cells (CD14^−^/CD11c^+^/4G2^+^) after treatment with NAR. (**D**) The frequency of ZIKV-infected hmdDCs (CD14^−^/CD11c^+^/4G2^+^) after treatment with NAR. (**E**) The viral titers detected by foci-forming immunodetection assay (FFU_C6/36_/mL) in hmdDCs culture supernatant after 24 hours of infection. Data represent each donor measure and were evaluated by paired one-way ANOVA and Tukey’s post-test (*p < 0.05 *vs* ZIKV-infected and untreated cells).

Also, due to the role of dendritic cells in the immune response and its highly susceptibility to ZIKV infection we decide to test the antiviral activity of NAR in human monocyte-derived dendritic cells (hmdDCs)^41–43^. First, it was shown that the concentration of 125 µM of NAR was not toxic to hmdDCs and would be used as the MNTC (Fig. 7B). Next, it was demonstrated that NAR (125 µM) effectively reduced the number of ZIKV-infected hmdDCs as well as the viral titers in cell culture supernatants (Fig. 7C–E). Altogether, the results demonstrated that NAR is able to impair the infection of hmdDCs, a key target cell for ZIKV infection and for the immune system function^41,44^.

## Discussion

The Zika virus epidemic in Brazil (2015) was associated with new clinical syndromes, such as *Guillain-Barré* and neurological defects in children born from infected women^5,9–11^. Despite the many efforts to reduce the morbidity and mortality associated with this infection, to date, neither a vaccine nor an antiviral drug has been licensed for use in humans^45^.

Several different compounds were already tested against ZIKV, mostly *in vitro*^12,27,28,46^. Additionally, it was already demonstrated that flavonoids could represent a source of active compounds against flaviviruses^18–26,47–49^. Recently, NAR was shown to impair the replication of dengue viruses in human cells^18^.

The cytotoxicity of NAR diverges, depending on the given cell line^48,50^. A 250 μM concentration of NAR could be used in human hepatoma cell culture (Huh7.5) without toxic effects^18^. Here, the toxicity of NAR to A549 was demonstrated to be concentration-dependent, and 125 µM (MNTC) was considered safe. For the other assayed cell lines or primary cells (Huh7.5, A172, hmdDCs and NKX2-5^eGFP/w^hESC), the same concentration was also non-toxic. However, higher cytotoxicity has been previously observed for primary human monocytes treated with NAR^18^. *In vivo*, NAR seems to be well absorbed and tolerated by mice, and also protects rats from dimethylnitrosamine-induced liver damage^51,52^.

The antiviral effect of flavonoids has been tested against numerous viruses such as herpes simplex viruses, hepatitis B and human cytomegalovirus^14,53,54^. Additionally, the flavanone NAR presents antiviral activity against viruses from the *Flaviviridae* family such as HCV, dengue and yellow fever virus strain 17D^17,18,55^. It appears that some flavonoids are able to interact with the NS2B-NS3 protease of ZIKV through chemical structures, and they play important roles in the inhibition activities of this protease^28^.

Here, four different techniques showed that NAR was able to impair the ZIKV infection in A549 cells. Additionally, we observed that NAR performed better than IFN-α 2A in reducing the virus titer in the cell culture supernatant. Thus, it seems that IFN-α 2A reduces the number of ZIKV-infected A549 cells, despite having lower impact on the secretion of viable ZIKV particles. The same phenotype was observed for A172 and Huh7.5 cells treated with IFN-α 2A. Goebel *et al*.^56^, have shown that high concentration (500 IU/mL) of type I IFN is needed to reduce 1–2 log of ZIKV RNA in Vero cell culture supernatant. Finally, this effect seems to be specific for ZIKV infection, once IFN-α 2A was able to reduce the secretion of viable dengue virus particles in cell culture supernatants to the same extent as the percentage of Huh7.5-infected cells^18^. Thus, here the IFN-α 2A was used as an *in vitro* ZIKV-treatment control as already demonstrated in the literature^46^. Once type I IFN has a broad mechanism to control viral infection, through the expression of hundreds of ISGs with antiviral activity^57^, we do not intend compare the anti-ZIKV effect of NAR with type I IFN.

Also, using two different techniques it was suggested that NAR does not present virucidal effect against ZIKV. In agreement, previous report shows that NAR was not virucidal to the four-dengue virus serotypes^18^.

Furthermore, NAR was able to inhibit the infection of Huh7.5 cells by the four dengue serotypes^18^. However, when using the dengue-2 New Guinea C strain, Zandi *et al*.^48^ were not able to show infection inhibition. Additionally, it was shown that the flavonoid isoquercitrin present anti-ZIKV activity for both African- and Asian-lineages of the virus^23^. In this context, we tested the antiviral activity of NAR against distinct ZIKV recent clinical isolates from Asian-lineage obtained from patients in Brazil, and from African-lineage isolated from a Rhesus monkey in Uganda^1,58,59^. The results showed that the anti-ZIKV activity of NAR is lineage-independent, although a higher reduction of infection was observed for Asian-lineages of ZIKV when compared to the African-lineage. Intrinsic differences in the pathogenicity and virulence of African- and Asian-lineage of ZIKV were already demonstrated^37,60^. While those differences could account for differences in clinical presentation needs to be better defined. However, Asian-lineage seems to infect cells at lower rate, produces less virus and trigger poor early cell death compared to the more virulent African-lineage^37^, which could help to explain the observed results.

An additional aspect of anti-ZIKV activity of NAR that should be taken into consideration is the cell line used in the experiments. Anti-ZIKV activity of NAR seems to be cell-line dependent, once ZIKV-infection of human embryonic stem cell line was not affected neither by NAR nor by IFN-α 2A. Otherwise, for three cell lines used and a primary human cell (hmdDCs) NAR seems to be able to impair ZIKV infection. It was demonstrated that the absorption and metabolism of the flavonoids would vary between cell lines^61^. This associated with differential subcellular distribution and efficiency of NAR accumulation inside the cells might lead to differential cell-line responses observed^62^.

To determine the stage of the ZIKV life cycle that is affected by NAR, a time-of-drug-addition experiment was performed^18,38^. The data indicate that NAR acts between replication and virus assembly, as already shown for the dengue and Chikungunya viruses^18,63^. In addition, the antiviral effect of NAR was evident even when NAR treatment was postponed for 24 hours after infection, and it was similar to IFN-α 2A. Similarly, treatment of ZIKV-infected Vero cells with 6-methylmercaptopurine riboside (6MMPr) 72 hpi impairs viral RNA production and demonstrated the potential use as antiviral compound^64^. Furthermore, some treatments available for flaviviruses, like IFN-α 2A for hepatitis C present several adverse effects, such as pain and depression^65^. Thus, the fact that NAR also inhibit acute inflammation and reduce pain, would represent an additional advantage of using NAR for treating ZIKV infected patients^31,32,65,66^.

The inhibition of viral RNA metabolism occurs through flavonoid binding, as shown for the flavonol called kaempferol against Japanese encephalitis virus^49^. Furthermore, flavonoids seems to impair the activity of proteins related to replication, such as the NS2B-NS3 complex of dengue and ZIKV^26,28^. Flavonoids are non-competitive inhibitors of the viral protease in both dengue and ZIKV^26–28^. A reduction in the possible hydrogen bonds of apigenin, as shown by molecular docking with the viral NS2B-NS3 protease, is consistent with the experimental data. The concentration of apigenin necessary to inhibit half the maximum activity of the protease is 43 times higher than that of myricetin, which can establish more hydrogen bonds^27,28^. According to the molecular modeling data, NAR is able to interact with the viral protease in the same way as other flavonoids, which are known allosteric inhibitors of this protein. Thus, we propose that the molecular target of NAR is the NS2B-NS3 protease. However, experimental validation is needed to confirm this hypothesis.

## Conclusions

Here, we used different techniques to show that NAR presents anti-ZIKV activity. *In vitro* NAR was effective against distinct ZIKV lineages (Asian and African) and seems to act during the late phase of the viral life cycle, probably as a non-competitive inhibitor of NS2B-NS3 protease. NAR keeps its antiviral activity even when it is added several hours post-infection, supporting the idea that NAR can target virus replication. Altogether, the results demonstrated that NAR could be a suitable candidate for ZIKV therapy. Finally, further studies should be undertaken to improve the understanding of the *in vitro* antiviral activity of NAR and to establish whether it is effective *in vivo*.

## Methods

### Cells, Zika virus and naringenin

*Aedes albopictus* C6/36 (ATCC: CLR-1660) mosquito cells and Human-derived hepatoma cells (Huh7.5; ATCC PTA-8561) were grown as previously described^18^. Human A549 lung epithelial cells (ATCC CCL-185) were grown in Dulbecco’s Modified Eagle Medium consisting of an F-12 nutrient mixture (DMEM/F-12), supplemented with 25 µg/mL of gentamicin and 7% fetal bovine serum (FBS). Human gliobastoma cell line (A172; ATCC^®^ CRL-1620^TM^) was grown in DMEM high glucose supplemented with 100 IU/mL penicillin, 100 µg/mL streptomycin and 10% FBS. Human embryonic stem cell line (NKX2-5^eGFP/w^hESC) was seeded on matrigel-coated culture plates using the mTeSR^TM^1 medium (StemCell Technologies) and cultured as previously described^67^. The NKX2-5^eGFP/w^hESC cells were kindly provided by Dr. David Elliot from the Murdoch Childrens Research Institute (MCRI), Australia. A549, A172, Huh7.5 and NKX2-5^eGFP/w^hESC were all cultured at 37 °C in a humidified, controlled 5% CO_2_ atmosphere. Human monocyte-derived dendritic cells (hmdDCs) were generated from peripheral blood monocytes from healthy donors as already described^68^, however using 25 ng/mL of human recombinant IL-4 (rIL-4) and 12.5 ng/mL of human recombinant GM-CSF (rGM-CSF) (PeproTech, Rocky Hill, NJ, United States). The study with human cells from healthy donors was approved by Committee of Human Experimentation from Fundação Oswaldo Cruz (Fiocruz) under the number CAAE: 60643816.6.0000.5248. All reagents, unless specified, were from Thermo Fisher Scientific (Grand Island, New York, USA).

Zika viruses were isolated from serum of infected patients in Northeast (2015) (ZV BR 2015/15261; ZV BR 2015/15098) and South Brazil (2016) (ZV BR 2016/16288). Since our laboratory is a Reference Center for the Diagnosis of Emerging Viruses of the Brazilian Ministry of Health, we obtained the waiver from the Brazilian National Ethics Committee of Human Experimentation for the written informed consent to work with these samples. However, the study with human serum samples was approved by Fundação Oswaldo Cruz (Fiocruz) and the Brazilian National Ethics Committee of Human Experimentation under the number CAAE: 42481115.7.0000.5248. Finally, the study is in compliance with all the ethical principles of the Brazilian National Ethics Committee of Human Experimentation. Additionally, the laboratory-adapted ZIKV strains ZIKV PE243^59^ and ZIKV MR766^1^ were tested. Viral stocks were grown in C6/36, clarified by centrifugation and titrated using a foci-forming immunodetection assay in C6/36.

Naringenin (NAR; ≥ 98% purity) was purchased from Santa Cruz Biotechnology (Dallas, TX, USA), prepared in a 50 mM stock solution in dimethyl sulfoxide (DMSO, Sigma-Aldrich, St. Louis, MO, USA) and then diluted to its final concentrations in DMEM/F-12 supplemented with 100 IU/µg/mL penicillin/streptomycin and 10% FBS (Thermo Fisher Scientific, Grand Island, New York, USA).

### Cell viability assay

A549 cell viability following NAR treatment was tested by Annexin-V/7-AAD (BD Biosciences, San Jose, CA, USA) cell staining procedure. A549 cells (1 × 10^5^ cells/well in 24 well plates) were incubated for 18 hours and treated with different concentrations of NAR (2,000, 1,000, 500, 250, 125 and 62.5 µM) or 1% DMSO (vehicle). The cells were maintained for 48 hours. After incubating, the cells were trypsinized, labelled with Annexin-V (FITC) and 7-AAD, and analyzed by flow cytometry (FACS) (FACS Canto II; BD Biosciences, San Jose, CA, USA). The viable A549 cells were defined as double-negative for Annexin-V^−^/7-AAD^−^ and the resulting data were used to determine the maximal non-toxic concentration (MNTC) of NAR. Additionally, the concentration that inhibited viability in 50% of the cells (CC_50_) was obtained using nonlinear regression and a sigmoidal concentration-response curve (GraphPad Prism, La Jolla, CA, USA). The same protocol was used to determine the MNTC of NAR for Huh7.5, A172, NKX2-5^eGFP/w^hESC. For hmdDCs the expression of Annexin-V/7-AAD and the establishment of MNTC were performed after 24 h in culture. Finally, an immunofluorescence assay was performed to confirm that 125 μM of NAR did not affect A549 cells viability. Briefly, A549 cells (1 × 10^4^ cells/well in 96-well plates) were treated with NAR (125 μM) in DMEM-F12 for 48 hours. The cells were than fixed and permeabilized with methanol:acetone (v/v) and cell nuclei was stained with DRAQ5 (Thermo Fisher Scientific, Grand Island, New York, USA). The cell nuclei count after treatment with NAR were determined by an Operetta High-Content Imaging System using Harmony High Content Imaging and Analysis software (4 different fields and 20x magnification; Thermo Fisher Scientific, Rockford, IL, USA).

### Antiviral activity assays

The antiviral activity of NAR was assessed using immunofluorescence, FACS, one-step reverse transcriptase real time polymerase chain reaction (RT-qPCR) and foci-forming immunodetection assays. First, the A549 cells (1 × 10^5^ cells/well in 24 well plates) were incubated for 18 hours. The cells were then infected with ZIKV at a multiplicity of infection (MOI) of 0.1 for 90 minutes, washed and the MNTC of NAR (125 μM) was added. After a 48 hours incubation, the cell culture supernatants were removed and stored at −80 °C for virus titration in C6/36. Moreover, the cells were detached and stained for FACS using a 4G2-FITC monoclonal antibody (anti-E protein of flavivirus; ATCC® HB-112™) as previously described^18^. A BD FACS Canto II was used to quantify the number of 4G2-positive cells. Additionally, RNA extracted from the cell pellet (RNeasy Mini Kit, Qiagen, Hilden, Germany) was used to quantify the ZIKV NS2B genes using a RT-qPCR^69^. In brief, 5 µg of RNA was used to amplify the ZIKV NS2B gene using specific primers (10 pmol) and a probe (4 pmol). For normalization, an 18S gene amplification was performed using 10 pmol of the primers 18SF and 18SR. The final reaction volume was 20 μL. The gene modulation was determined by calculating the 2^−∆∆CT^ ^70^. The primer sequences and cycling conditions are listed in Table 1.

**Table 1:** Primer and probe sequences and the cycling conditions used during the RT-PCR assays.

Target gene	Primer name	Sequence (5′–3′)	Cycling
**Zika virus NS2B gene** ^70^	Zika4481	CTGTGGCATGAACCCAATAG	50 °C-30 min 95 °C-15 min 45 cycles 95 °C-15 s 60 °C-1 min.
	Zika4552c	ATCCCATAGAGCACCACTCC	
	Probe Zika4507c-FAM	CCACGCTCCAGCTGCAAAAGG	
**18S**	18SF	CACGGCCGGTACAGTGAA	42 °C-30 min 95 °C-10 min 40 cycles 95 °C-15 s 60 °C-30 s 72 °C-1 min
	18SR	CCCGTCGGCATGTATTAGCT	
**Zika virus envelope** (**E**) **gene**^72^	ZIKVENVF	GCTGGDGCRGACACHGGRACT	95 °C-2 min 35 cycles 95 °C-20 s 55 °C-20 s 72 °C-30 s Final elongation at 72 °C-7 min
	ZIKVENVR	RTCYACYGCCATYTGGRCTG	

For the immunofluorescence assay, the A549 cells (1 × 10^4^ cells/well in 96-well plates) were infected with MOI 0.1. After 90 minutes, the cells were washed and treated with NAR (125 μM) in DMEM-F12. After 48 hours, the cells were fixed and permeabilized with methanol:acetone (v/v) and the cell infection was analyzed after being labeled with 4G2 and goat anti-mouse Alexa Fluor 488 secondary antibody (Thermo Fisher Scientific, Grand Island, New York, USA). Digital images were taken from 4 different fields (20x magnification). The infection percentage was quantified in an Operetta High-Content Imaging System using Harmony High Content Imaging and Analysis software (Thermo Fisher Scientific, Rockford, IL, USA).

For all the techniques, mock (non-infected cells) and non-treated ZIKV-infected cells were used as controls. In addition, based on the anti-ZIKV activity of type I IFN *in vitro*^46^, interferon-α 2A (IFN-α 2A) (200 UI/ml; Blau Farmacêutica, Cotia, SP, Brazil) was used as a positive control for the antiviral treatment. Furthermore, concentration-response curves were obtained using a serial dilution starting from the MNTC of NAR. The concentration that inhibited 50% and 90% of the viral infection (IC_50_ and IC_90_, respectively) was obtained using a nonlinear regression, followed by a sigmoidal concentration-response (variable slope; GraphPad, La Jolla, CA, USA). Also, the selectivity index (SI = CC_50_/IC_50_) was calculated.

To access the anti-ZIKV activity of NAR on Huh7.5 (MOI 0.1), A172 and NKX2-5^eGFP/w^hESC (MOI 1) cells FACS and foci-forming immunodetection assays were employed as previously described for A549 cells using three different ZIKV-strains (ZV BR 2015/15261; ZIKV PE243 and ZIKV MR766).

The anti-ZIKV activity of NAR in hmdDCs was determined 24 hpi after infection with an MOI of 10 based on previously reported data^71^. For hmdDCs, the antiviral activity of NAR was analyzed 24 hpi, once it was demonstrated that both virus release and viral RNA loads increased over time reaching the peak at 24 hpi^43^.

### Foci-forming immunodetection assay

Viral titers in cell culture supernatant were determined by the foci-forming immunodetection assay in C6/36 cells (FFU_C6/36_/mL). Briefly, cell culture supernatants were 10-fold serially diluted in Leibovitz’s medium 15 (L-15) supplemented with 0.26% of tryptose and 25 µg/mL gentamicin and added to C6/36 (1 × 10^5^ cells/well in 24 well plates) in duplicate. After 1h30 min, the inoculum was removed and a CMC overlay media (L-15 supplemented with 5% SFB, 0.26% tryptose, 25 µg/mL gentamicin, 1.6% carboxymethylcellulose) was added and plates incubated at 28 °C. The immunostaining was performed after seven days using the mouse monoclonal Flavivirus group-specific antibody 4G2, followed by goat anti-mouse immunoglobulin conjugated to alkaline phosphatase (Promega, Madison, WI, USA), which was detected by adding a solution of NBT/BCIP (Promega, Madison, WI, USA) as a substrate. Foci was counted and expressed as FFU_C6/36_/ml.

### Virucidal Assay

A virucidal assay was performed as described^18^. In brief, 2 × 10^6^ FFU_C6/36_ of ZIKV (ZV BR 2015/15261) was incubated with NAR (125 μM) in the presence or absence of RNase A (150 μg/mL) (USB-Affymetrix; Santa Clara, CA, USA) at 37 °C for 1 hour. Zika virus RNA samples that were treated with RNase or left untreated were used as controls. After incubation, the amplification of ZIKV envelope gene was performed based on a one-step RT-PCR assay previously described^72^. The primer sequences and cycling conditions are available in Table 1. Additionally, 2 × 10^6^ FFU_C6/36_ of ZIKV was incubated with NAR (125 μM) for 1 h at 37 °C and residual infectivity of ZIKV was assessed by foci-forming immunodetection assay in C6/36 as described above.

### Time of drug-addition assay

The treatment of the infected cells at different moments of the infection could indicate in each step of the virus life cycle the compound act. Thus, the time of drug-addition assay was performed as previously described^18,38^. Initially, the A549 cells (1 × 10^5^ cells/well in 24 well plate) were submitted to different treatment regimens: (1) infected and left untreated (positive control of infection); (2) treated with NAR (125 μM) for 1.5 h prior to ZIKV infection; (3) treated with NAR during ZIKV infection; (4) treated with NAR during and after ZIKV infection; (5) treated with NAR after ZIKV infection; or (6) treated with IFN-α 2A (200 UI/ml) after ZIKV infection as a control of treatment (Fig. 5A). After incubation the number of infected cells (FACS), and the virus titers at cell culture supernatant was performed for each different treatment.

Furthermore, the cells were infected with ZIKV MOI 0.1 and treated with NAR (125 μM) at different time points after ZIKV infection, namely as 0, 1, 2, 4, 6, and 24 hours post-infection (hpi) (Fig. 6A). The same experimental conditions were tested using IFN-α 2A (200 UI/ml) as a positive control for the antiviral treatment. The number of infected cells was determined by FACS, and the virus titers at cell culture supernatant was performed to evaluate the impact of NAR treatment at different time points on A549-infected cells.

### Molecular docking assay of NAR

Docking calculations were performed with AutoDock Vina^73^ with the value of the exhaustiveness parameter set to 1000. The 3D structure of all the compounds was initially optimized with MOPAC software^74^ using the semi-empirical Hamiltonian PM7^75^ in a vacuum. The receptor structure was the crystallographic structure of the NS2B-NS3 protease from the ZIKV in a complex with the peptide PDB ID 5GPI^76^. The peptide and water molecules from the experimental structure were removed and chains A and B were used as representatives of the NS2B and NS3pro subunits, respectively. Hydrogens were added using GROMACS 2016.3^77^. The search volume for the docking was focused on the allosteric cavity that was previously identified for the dengue virus protease^78^. Pictures were generated with the open source version of PyMOL 1.8.6.0 (Schrödinger, LLC). The hardware was provided by a Brazilian biotech startup called Mining Information for You (MI4U) and was composed of one machine with 32 physical cores that was hosted by the Google Cloud Platform.

### Data analysis

The statistical analysis consisted in a one-way ANOVA and Tukey’s post-test with a significance of *p* < 0.05, and it was performed with Prism software (GraphPad version 6.0; La Jolla, CA, USA). Flow cytometry data were analyzed using the FlowJo software version 10 (Tree Star Inc., USA).

## Supplementary information

Supplementary Material
