# Supplementary material for: The citrus flavonoid naringenin impairs the in vitro infection of human cells by Zika virus
Source: Sci Rep. 2019 Nov 8;9:16348. doi: 10.1038/s41598-019-52626-3 (PMC6841724; doi:10.1038/s41598-019-52626-3)
Supplement: Supplementary file 1 — Supplementary Material [file 41598_2019_52626_MOESM1_ESM.pdf]

Supplementary Material

The citrus flavonoid naringenin impairs the *in vitro* infection of human cells by Zika virus

Allan Henrique Depieri Cataneo[1,a], Diogo Kuczera [1,a], Andrea Cristine Koishi[1], Camila Zanluca[1], Guilherme Ferreira Silveira[1], Thais Bonato de Arruda[1], Andréia Akemi Suzukawa[1], Leandro Oliveira Bortot[2], Marcelo Dias-Baruffi[3], Waldiceu Aparecido Verri Jr[4], Anny Waloski Robert[5], Marco Augusto Stimamiglio[5], Claudia Nunes Duarte dos Santos[1], Pryscilla Fanini Wowk[1,*], Juliano Bordignon[1,*]

*** Correspondence:** Pryscilla Fanini Wowk and Juliano Bordignon
E-mail: pryscilla.wowk@fiocruz.br; juliano.bordignon@fiocruz.br

1.1 Supplementary Table

Supplementary Table 1: Primer and probe sets used for the discrimination between Asian- and African-lineage of Zika virus

Primer/Probe	Sequence (5'-3')	Genome Position	Lineage specificity	Reference
ZIKV 1086	CCGCTGCCCAACACAAG	1086-1102	Asiatic + African	Lanciotti et al., 2008 [35]
ZIKV 1162c	CCACTAACGTTCTTTTGCAGACAT	1162-1139		
ZIKV 1107-FAM	AGCCTACCTTGACAAGCAGTCAGACACTCAA	1107-1137		
Zika 4481	CTGTGGCATGAACCCAATAG	4434-4453	Asiatic	Bingham et al., 2016 [36]
Zika 4552c	ATCCCATAGAGCACCACTCC	4524-4505		
Zika 4507c-FAM	CCACGCTCCAGCTGCAAAGG	4479-4460		

1.2 Supplementary Figures

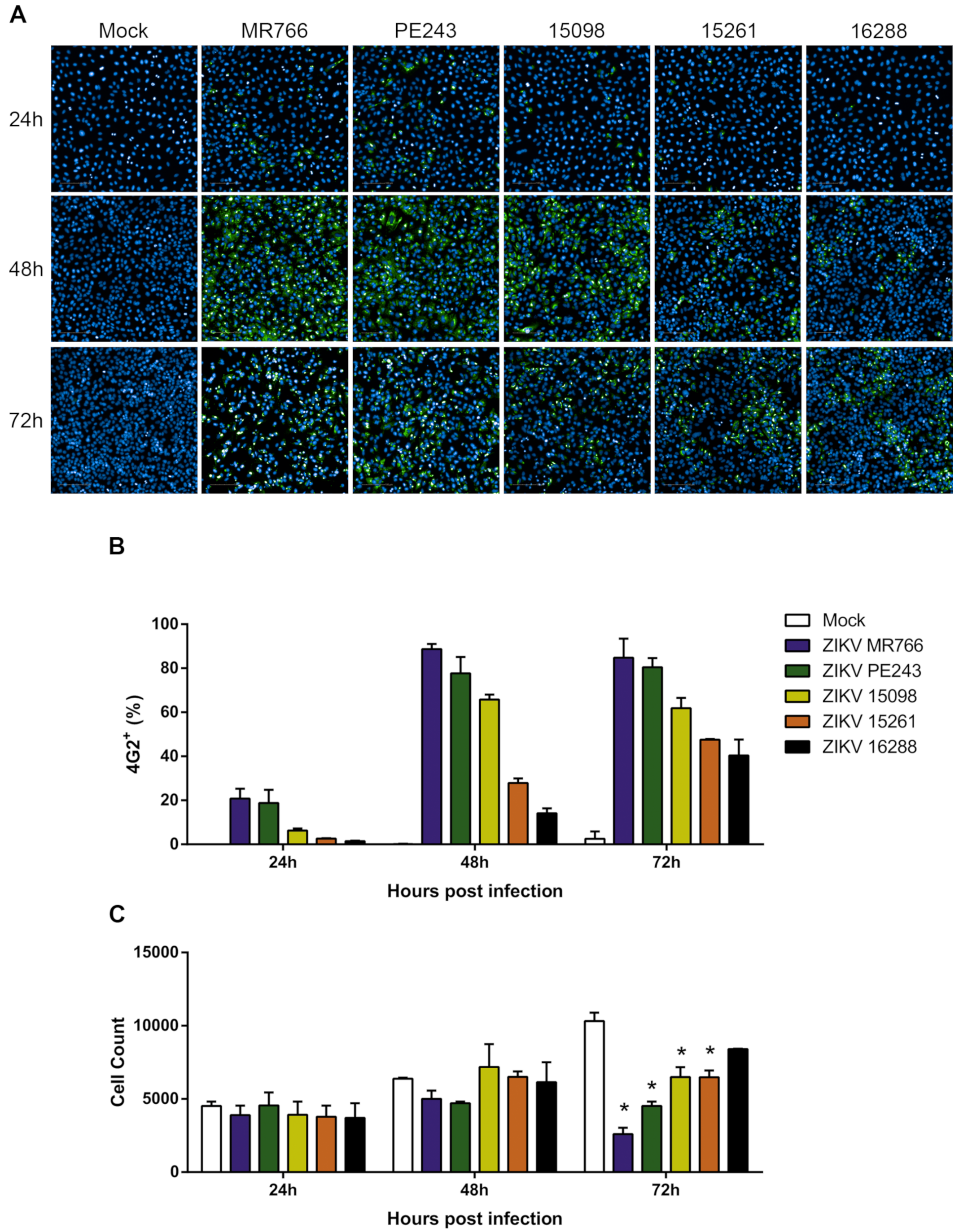

Supplementary Figure 1. Zika virus growth curve on A549 cells. A549 cells (1x10[4] in 96-well plate) were infected with five strains of ZIKV (ZIKV MR766, ZIKV PE243, ZV BR 2015/15098, ZV BR 2015/15261 or ZV BR 2016/16288; MOI 0.1). After 24, 48 and 72 hours post-infection, the numbers of infected A549 cells were quantified using a high-content imaging assay (20X magnification; *Operetta High-Content Imaging System from*

PerkinElmer). **(A)** Representative imaging of A549 cells obtained by the Operetta System showing cell nuclei (blue; counterstained with DAPI), and anti-flavivirus E protein monoclonal antibody (green; 4G2 staining) plus goat anti-mouse Alexa Fluor 488 secondary antibody. **(B)** The average number of infected-A549 cells. **(C)** The average number of cells (cell nuclei count) presented in culture in each experimental condition and time-point. Data are the representative of 9 different fields at each well (*$p<0.05$ ZIKV-infected *vs* MOCK).

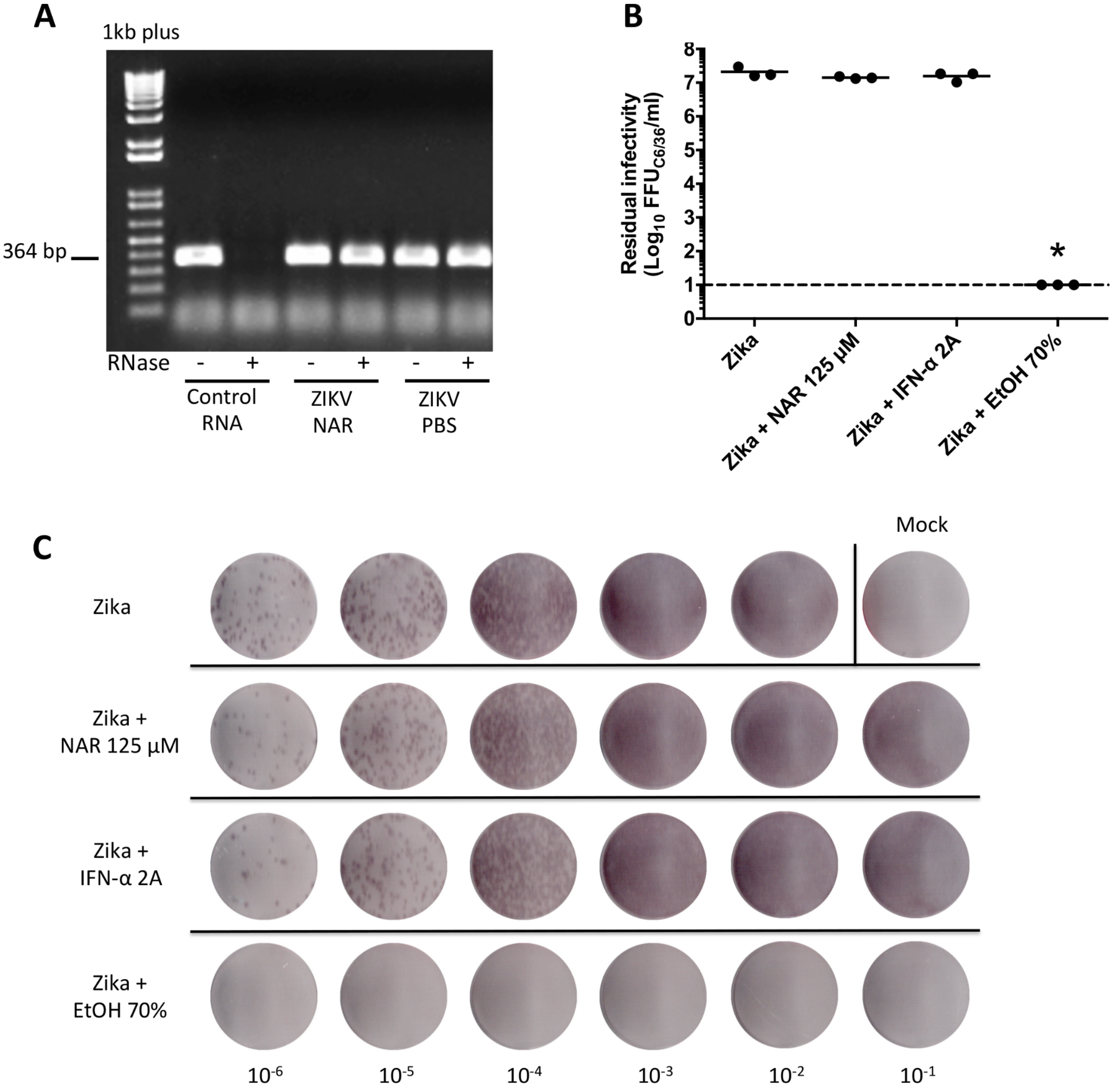

Supplementary Figure 2. NAR does not present virucidal activity against Zika virus. The ZV BR 2015/15261 was incubated with 125 µM of NAR in the presence or absence of RNase. Following a 1h incubation, the RNA samples were extracted and tested for the amplification of a fragment of the envelope gene (E) from the ZIKV using the RT-PCR technique. **(A)** An agarose gel (1%) stained with ethidium bromide demonstrated the amplification of a 364 bp fragment of the E gene from ZIKV. The figure shows a representative result from three independent experiments. As a control of the amplicon size 1kb Plus (ThermoFisher) was used. The original gel is shown below. **(B)** The ZV BR 2015/15261 was incubated with 125 µM of NAR for 1 hour. After the incubation, the residual infectivity was measured by titration in C6/36 cells as stated in material and methods ($FFU_{C6/36}$/mL). As a control, ZV BR 2015/15261 was incubated for the same time with IFN-α 2A or 70% ethanol. **(C)** Representative picture of a titration of ZV BR 2015/15261 in C6/36 cells after different treatments. Data represent three independent experiments performed in technical triplicate and analyzed by one-way ANOVA followed

by Tukey´s Multiple Comparison Test (*p<0.05 *vs* ZIKV-infected and untreated cells; *vs* ZIKV-infected and treated with NAR or IFN-α 2A).

Original agarose gel image for Supplementary Figure 2A.

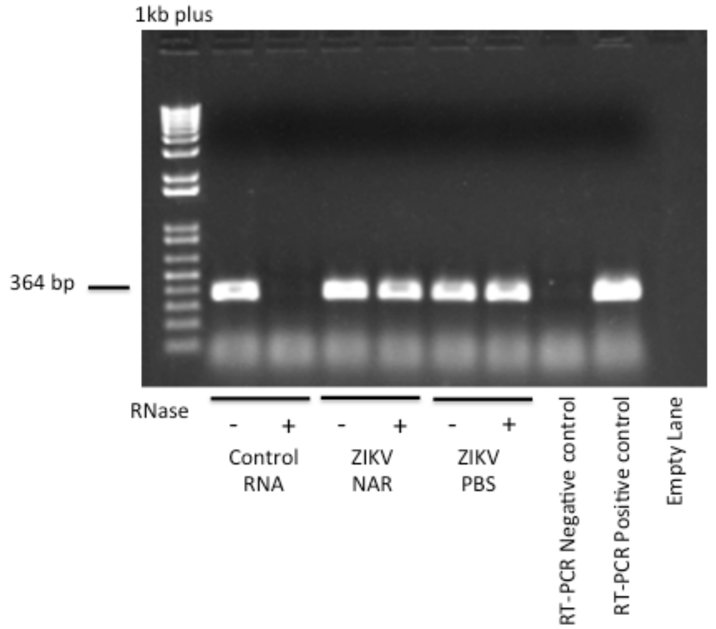

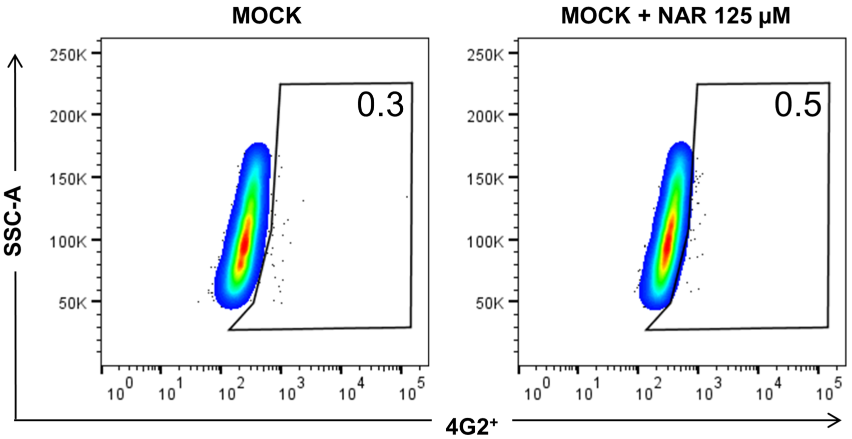

Supplementary Figure 3. NAR does not affect A549 autofluorescence during flow cytometry. The A549 cells were treated with NAR (MOCK + NAR 125 μM) or left untreated (MOCK). The impact of NAR on A549 cell autofluorescence was analyzed by flow cytometry after staining with anti-flavivirus E protein monoclonal antibody (4G2-FITC). The representative results from three independent experiments, with each one in technical triplicate.

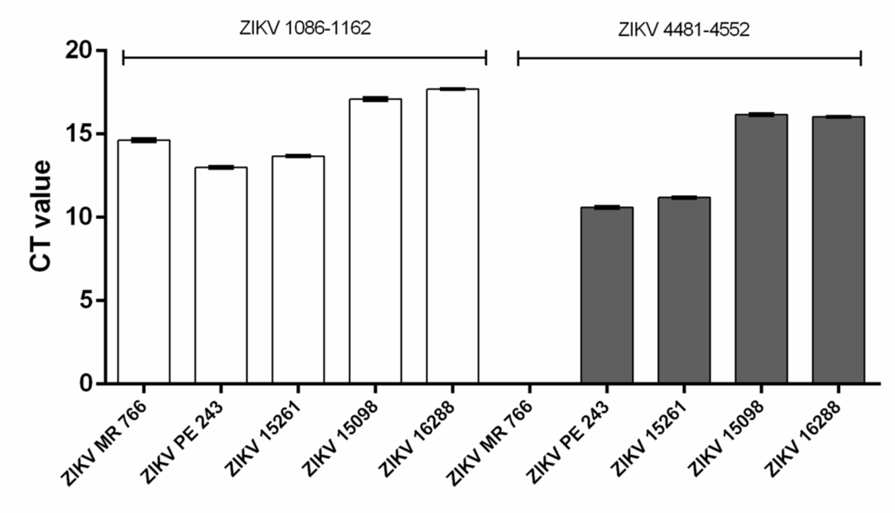

Supplementary Figure 4. Real time PCR for ZIKV lineage determination. Viral RNA was extracted from the five strains of ZIKV cell culture supernantants using QIAamp Viral RNA Mini Kit (Qiagen, Hilden, Germany) and used for real time PCR. Primers and probes for discrimination between Asian- and African-lineages of ZIKV were used following Lanciotti et al., 2008 and Bingham et al., 2016. The threshold cycle for each amplification was used to confirm the amplification of each RNA sample with different primer and probes sets. Result represent the mean plus standard deviation of three technical replicate for one real time PCR amplification.

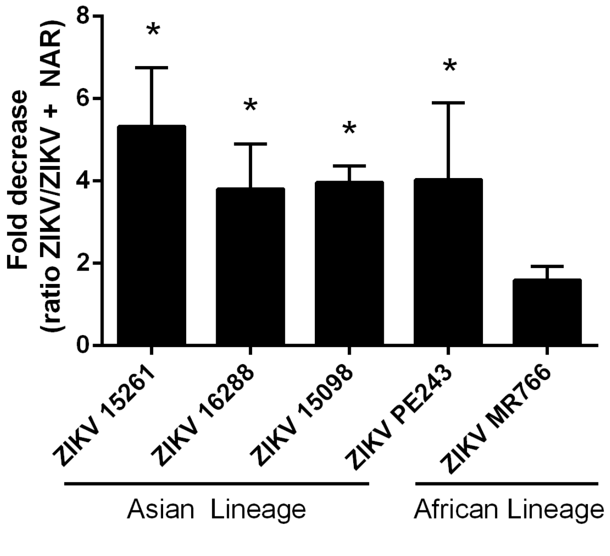

Supplementary Figure 5. NAR treatment was more effective against Asian than African lineages of ZIKV. The data from flow cytometry of Figures 2 and 4 were used to calculate the ratio between the frequency of untreated ZIKV-infected cells and the frequency of NAR-treated ZIKV-infected cells. Data represent three independent experiments performed in technical triplicate and analyzed by one-way ANOVA followed by Tukey´s Multiple Comparison Test (*p<0.05 *vs* ZIKV MR766).

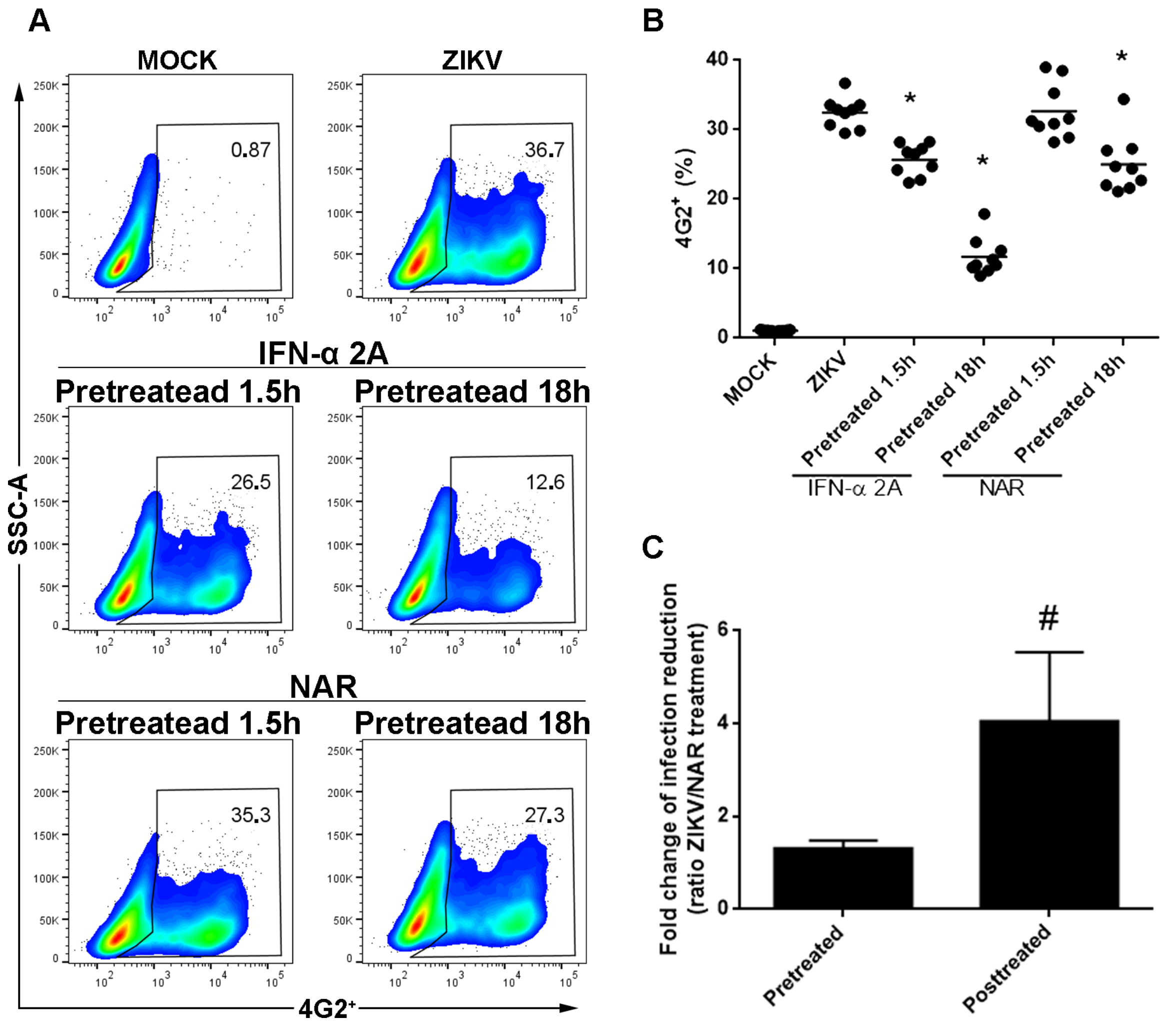

Supplementary Figure 6. Comparison of anti-ZIKV activity of NAR pretreatment and posttreatment on A549 cells. The A549 cells were pretreated with NAR (125 µM) for 1.5h or 18h prior to ZIKV infection with 0.1 MOI of ZIKV (ZV BR 2015/15261 strain). Pretreatment with IFN-α 2A (200 UI/ml) was used as control. **(A)** Representative density plot showing the frequency of infected cells ($4G2^+$) in each experimental condition. **(B)** Frequency of NAR pretreated ZIKV-infected cells ($4G2^+$). **(C)** Comparison of ZIKV-infected cells ($4G2^+$) frequency between NAR pretreatment (18h) and posttreatment. Data from three independent experiments in technical triplicate that were analyzed by one-way ANOVA followed by Tukey´s Multiple Comparison Test (*p<0.05 *vs* ZIKV-infected cells; #p<0.05 *vs* NAR pretreated cells).

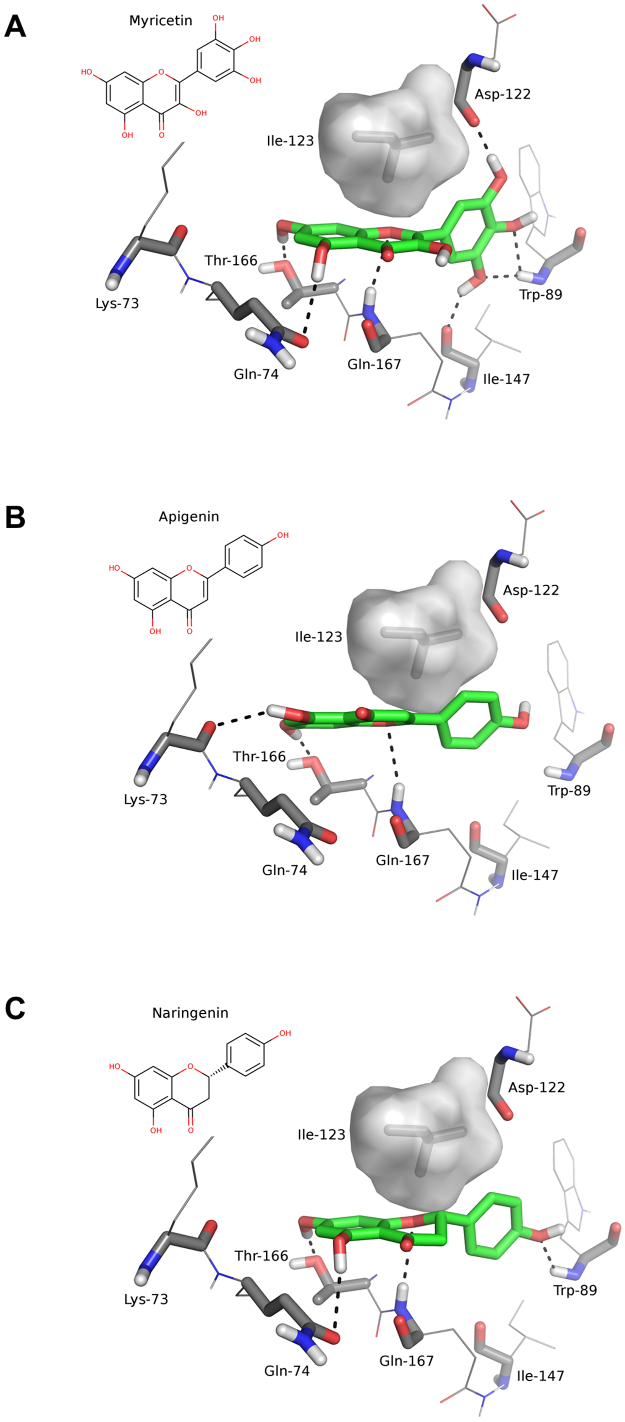

Supplementary Figure 7. Proposed molecular interaction mode between the Zika Virus protease with different flavonoids. (A) Myricetin**, (B)** Apigenin**, (C)** Naringenin**.** The relevant residues of the protease are labelled. Hydrogen, Nitrogen and Oxygen atoms are displayed in white, blue and red, respectively. Carbon atoms from the protease and flavonoids are shown in gray and green, respectively. Ile-123 is shown in surface representation to emphasize its involvement in hydrophobic contacts with the flavonoids. Hydrogen bonds are shown as dashed lines.

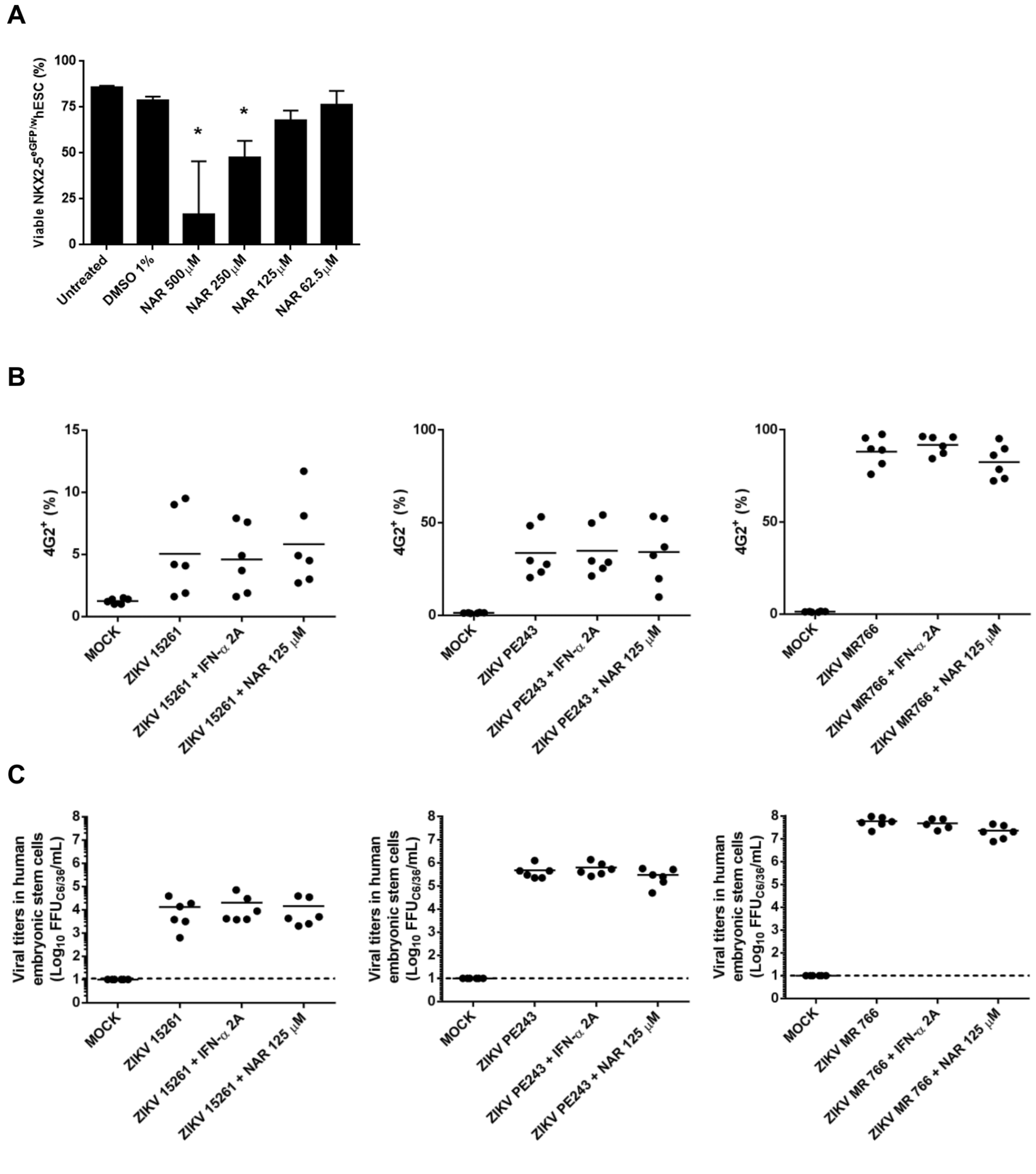

Supplementary Figure 8. Anti-Zika virus activity of NAR in human embryonic stem cells. The NKX2-5$^{eGFP/w}$hESC cells were treated with different concentrations of NAR (500 to 62.5 μM) over 48 hours and cell viability analyzed by flow cytometry by Annexin-V and 7-AAD staining. Also, cells were infected with ZIKV MOI 1 (ZV BR 2015/15261; ZIKV PE243 and ZIKV MR766) and treated with NAR (125 μM) and analyzed by flow cytometry. **(A)** Frequency of viable NKX2-5$^{eGFP/w}$hESC cells (Annexin-V$^-$/7-AAD$^-$) after treatment with different NAR concentrations. **(B)** Flow cytometry analyses showing the frequency of ZIKV-infected cells stained with anti-E monoclonal antibody (4G2$^+$). **(C)** Viral titers in cell culture supernatant detected by focus-forming assay (FFU$_{C6/36}$/mL). DMSO was used as a control and vehicle for the preparation of NAR stock. IFN-α 2A (200

IU/mL) was used as a positive anti-viral control, and the non-infected cells (MOCK) were used as a negative control. Results from three independent experiments performed in technical triplicate and analyzed by one-way ANOVA followed by Tukey´s Multiple Comparison Test (*$p<0.05$ *vs* ZIKV infected and untreated cells).

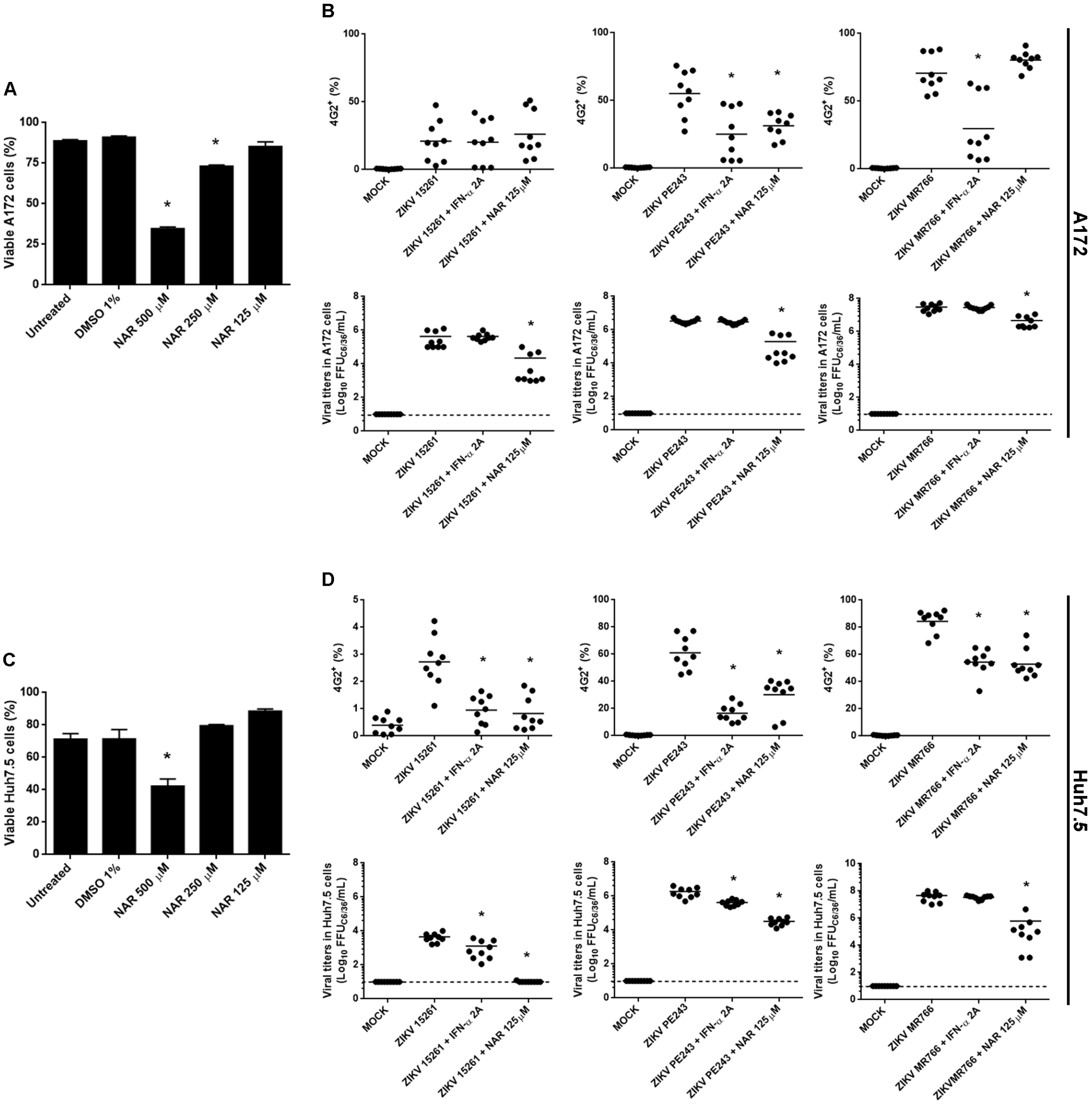

Supplementary Figure 9. Anti-Zika virus activity of NAR in different cell lines. A172 (MOI of 1) and Huh7.5 (MOI 0.1) cells were infected with ZIKV (ZV BR 2015/15261; ZIKV PE243 and ZIKV MR766) and treated with NAR (125 μM). After 48 hours, the cells were harvested and stained for a flow cytometry assay using anti-flavivirus E protein monoclonal antibody ($4G2^+$) and viral titers in cell culture supernatant were determined using foci-forming immunodetection assay ($FFU_{C6/36}$/mL). IFN-α 2A (200 IU/mL) was used as a positive anti-viral control, and the non-infected cells (MOCK) were used as a negative control. **(A)** The frequency of viable A172 cells (Annexin-V^-/7-AAD^-). **(B)** Frequency of ZIKV-infected A172 cells ($4G2^+$) and viral titers ($FFU_{C6/36}$/mL) in cell culture supernatant after 48 hours of infection. (**C)** Frequency of viable Huh7.5 cells (Annexin-V^-/7-AAD^-). **(D)** Frequency of ZIKV-infected Huh7.5 cells ($4G2^+$) and viral titers ($FFU_{C6/36}$/mL) in cell culture supernatant after 48 hours of infection. Results from 3 independent experiments performed in technical triplicate and analyzed by one-way

ANOVA followed by Tukey's Multiple Comparison Test (*$p<0.05$ *vs* ZIKV-infected and untreated cells).
